# The Role of Rigid Residues in Modulating TEM-1 β-Lactamase Function and Thermostability

**DOI:** 10.3390/ijms22062895

**Published:** 2021-03-12

**Authors:** Bethany Kolbaba-Kartchner, I. Can Kazan, Jeremy H. Mills, S. Banu Ozkan

**Affiliations:** 1 School of Molecular Sciences, Arizona State University, Tempe, AZ 85281, USA; bkolbaba@asu.edu; 2 The Biodesign Center for Molecular Design and Biomimetics, Arizona State University, Tempe, AZ 85281, USA; 3 Center for Biological Physics, Arizona State University, Tempe, AZ 85281, USA; john.kazan@asu.edu; 4 Department of Physics, Arizona State University, Tempe, AZ 85281, USA

**Keywords:** protein dynamics, allostery, molecular dynamics, protein engineering, β-lactamases

## Abstract

The relationship between protein motions (i.e., dynamics) and enzymatic function has begun to be explored in β-lactamases as a way to advance our understanding of these proteins. In a recent study, we analyzed the dynamic profiles of TEM-1 (a ubiquitous class A β-lactamase) and several ancestrally reconstructed homologues. A chief finding of this work was that rigid residues that were allosterically coupled to the active site appeared to have profound effects on enzyme function, even when separated from the active site by many angstroms. In the present work, our aim was to further explore the implications of protein dynamics on β-lactamase function by altering the dynamic profile of TEM-1 using computational protein design methods. The Rosetta software suite was used to mutate amino acids surrounding either rigid residues that are highly coupled to the active site or to flexible residues with no apparent communication with the active site. Experimental characterization of ten designed proteins indicated that alteration of residues surrounding rigid, highly coupled residues, substantially affected both enzymatic activity and stability; in contrast, native-like activities and stabilities were maintained when flexible, uncoupled residues, were targeted. Our results provide additional insight into the structure-function relationship present in the TEM family of β-lactamases. Furthermore, the integration of computational protein design methods with analyses of protein dynamics represents a general approach that could be used to extend our understanding of the relationship between dynamics and function in other enzyme classes.

## 1. Introduction

Since the 1940s, β-lactam antibiotics, which target a key enzyme in bacterial cell wall biosynthesis, have been the antimicrobial weapon of choice in the war against bacterial infection [1]. The widespread use of β-lactams is likely a consequence of the fact that they are inexpensive to produce and have historically been effective in treating most infections. However, as the use of this class of antibiotics became more widespread, so too did the prevalence of β-lactamase enzymes, which hydrolyze the β-lactam ring and render the antibiotic nonfunctional [1]. Additionally, as new β-lactam antibiotics enter into clinical use, the remarkable adaptivity of β-lactamases complicates efforts to develop novel antibiotics that are resistant to degradation by this class of enzyme [2]. The TEM family of β-lactamases has been thoroughly studied to gain insight into the manner in which resistance is achieved [3,4,5,6,7]. Despite these efforts, we currently possess an incomplete understanding of the relationship between sequence and function in this enzyme class. A major challenge is that several mutations have been identified that have a significant influence on function, but which are highly distal from the enzyme active site [8]. In addition, even single point mutations (e.g., the well-characterized, M182T substitution), which have minimal effects on enzymatic function can drastically affect the protein’s thermostability [9,10]. Our inability to rationalize the manner in which these thoroughly studied mutations alter enzyme function is suggestive of an incomplete understanding of the sequence-function relationships present in β-lactamases. This in turn limits our ability to develop novel classes of antibiotics that are not substrates for these enzymes [11].

A possible explanation as to how mutations distal to the active site can still exert influence at a great distance is that they serve to reshape the inherent dynamics of the enzyme [12,13,14,15,16,17,18,19,20]. In a recent study, we explored this hypothesis in the TEM-1 β-lactamase using two in silico, dynamics-based metrics: the dynamic flexibility index (dfi) [16,21], which measures the mobility of each residue, and the dynamic coupling index (dci) [17,22], which assesses the coupling between distant residues [15]. Using these two metrics, we characterized TEM-1 and a set of ancestrally reconstructed TEM-1 variants that possess vastly distinct physical properties (i.e., thermostabilities) and functions (i.e., substrate specificity) despite having almost identical conformations [15,23,24,25]. A major finding of our previous study was that TEM-1 and its ancestral homologues possessed distinct dynamic profiles and that these differences in dynamics appeared to have profound effects on enzyme function. Namely, rigid residues that are distal from, but highly coupled to, residues in the active site appeared to have substantial effects on protein function [19,20,22,26]. One intriguing hypothesis that might explain these data is that rigid residues can serve as “hubs” of dynamic communication. This notion has also been validated in the context of disease-causing mutations in other proteins, ref. [27] in which mutations to rigid residues that are far from the active site are functionally deleterious [19,20,28,29].

More recently, we used both dfi and dci to analyze members of the TEM family that either arose in the clinic or were generated via directed evolution [19]. In this study, we observed that mutations known to confer resistance to non-native substrates (1) often occur at particularly rigid residues as judged by our dfi metric and (2) appear to allosterically modify the flexibility of catalytic residues within the active site as suggested by our dci metric [19]. Collectively, these studies support the hypothesis that rigid residues are of particular importance to the overall dynamics of proteins and may have a substantial impact on protein function if they are allosterically coupled to the active site. If our hypothesis is correct, mutations that alter the identity of allosteric rigid residues (or those in their vicinity) could have substantial effects on enzyme activity; however, the ability to thoroughly explore this hypothesis is challenging. Although extensive datasets comprised of clinically derived TEM family variants [30] and additional variants generated via directed evolution [25] exist, the serendipitous identification of proteins with multiple mutations in the vicinity of known rigid residues would be unlikely. One potential solution is to use computational protein design methods to specifically target mutations to regions of interest. A major benefit of this approach is the ability to “pre-screen” each combination of mutations in silico to exclude variants in which protein folding is not predicted to be energetically favorable.

In this work, computational protein design methods were used to alter the environments surrounding two residues that were identified as being rigid and highly coupled to the active site despite being separated from it by a great distance. Dynamic profiles of each designed protein (hereafter referred to as a “design”) were then generated and compared to that of an ancestrally reconstructed variant of TEM-1 (the “Gram-negative common ancestor” or GNCA), which possesses increased thermostability, but reduced activity against ampicillin relative to wild type TEM-1 [23]. Principal component analysis (PCA) was used to identify designs with dynamic profiles that were predicted to be more similar to GNCA than extant TEM-1, and five designs were characterized in the laboratory. All designs exhibited reduced activity against ampicillin relative to TEM-1, but an increase in thermostability was also observed. Reduced activity against ampicillin and increased thermostability relative to TEM-1 are both features of GNCA. Alternatively, when identical design protocols were applied to flexible residues that were not coupled to the active site, native-like catalytic abilities and thermostabilities were maintained. Finally, in an effort to further link dynamics to enzyme function, we developed a novel analytical approach termed the “dynamic distance analysis” (dda) that was applied retrospectively to our experimentally characterized proteins. The dda analysis appeared to capture functional differences between our designed proteins and could be a useful tool for dynamic profile analysis in future studies. Collectively, our results serve to further highlight the importance of allosteric rigid residues in regulating the dynamics of the TEM-1 β-lactamase.

## 2. Results and Discussion

### 2.1. Computational Analysis Using dfi and dci

**Figure 1:**
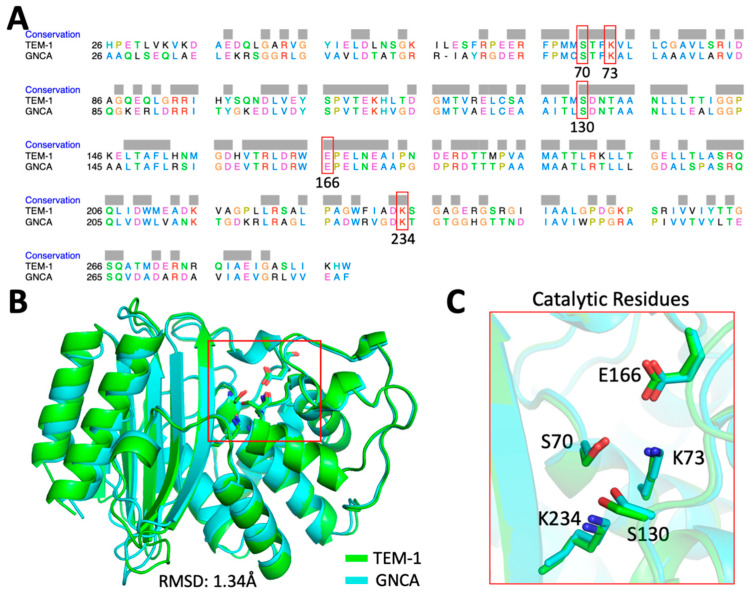
Differences in sequence and structure between TEM-1 and its ancestral variant GNCA. (**A**) Sequence alignment (Ambler numbering) [31] of TEM-1 and GNCA shows a 54% sequence identity; conserved active site residues are highlighted in red boxes. (**B**) The crystal structures of TEM-1 (PDB ID: 1btl, green [32]) and GNCA (PDB ID: 4b88, cyan [33]) are superimposed and the catalytic residues are shown as sticks within a red box. The low root-mean-square deviation (RMSD) indicates a high conservation of structure. (**C**) Active site residues in TEM-1 and GNCA are shown in green and blue sticks, respectively.

Our efforts to better understand the relationship between protein dynamics and function began by identifying a TEM-1 variant that could serve as a basis of comparison to the wild type protein. Recently, the putative sequences of ancestral TEM-1 were predicted using Bayesian bioinformatics [23]. Three ancestral TEM family homologues (the Gram-negative and Gram-positive common ancestor, PNCA; the Gram-negative common ancestor, GNCA, and enterobacteria common ancestor, ENCA) were observed to possess distinct physical and biochemical properties when characterized in the laboratory [23]. This is likely a consequence of the fact that these proteins are thought to have existed at different times in the evolutionary history of this enzyme [23]. We chose to focus our efforts on the ancestral homologue GNCA because its properties differ more substantially from TEM-1 than the other variants. Despite sharing > 50% identical residues (Figure 1A), nearly identical folds (1.3 Å root-mean-square deviation (RMSD) over all Cαs, Figure 1B), and conserved catalytic residues (Figure 1C), GNCA unfolds at a temperature that is ~35 °C higher than wild type TEM-1. Furthermore, GNCA appears to be a “substrate generalist” in that it possesses measurable (but reduced) activity against penam antibiotics (e.g., penicillin) relative to TEM-1, while simultaneously possessing a far greater ability to degrade cepham antibiotics (e.g., cefotaxime) [23].

It is difficult to rationalize the substantial differences in function and stabilities that are observed in GNCA and TEM-1 in light of the high sequence identity and structural similarities that exist for these proteins. Previous studies in our laboratory [15,19] suggested that the inherent dynamics of both TEM-1 and GNCA might play a role in regulating their functions. To further explore this, we analyzed the dynamic profiles of both proteins using two metrics developed in our group: The Dynamic Flexibility Index (dfi) and the Dynamic Coupling Index (dci). The dfi method [28,29,34] is based on Linear Response Theory and Perturbation Response Scanning [35] and calculates the resilience of a given residue to random force perturbations applied to other residues in the protein. A given amino acid’s dfi value is therefore related to the relative conformational entropy (i.e., flexibility) of that residue with respect to the rest of the protein. A high dfi value indicates high flexibility; conversely, a low dfi value indicates rigidity. The dci metric [17,19] is derived from the same theoretical origin as dfi and is used to quantify the degree to which two residues are dynamically coupled in terms of correlated motions. A high dci value between a pair of residues that do not interact directly indicates allosteric coupling and suggests that a perturbation to one residue will be transmitted to the other even over long distances. A low dci score implies a weak coupling between a residue pair, and no strong communication channel between them is expected.

When we applied the dfi and dci analyses to extant TEM-1 and a set of reconstructed ancestral homologues including GNCA [15,19], our analyses indicated that rigid residues (i.e., those with low dfi scores) that are highly coupled to the active site can contribute substantially to protein function. In this study, we hoped to further explore the importance of rigid residues to protein function by altering the identity of amino acids in their vicinity.

**Figure 2:**
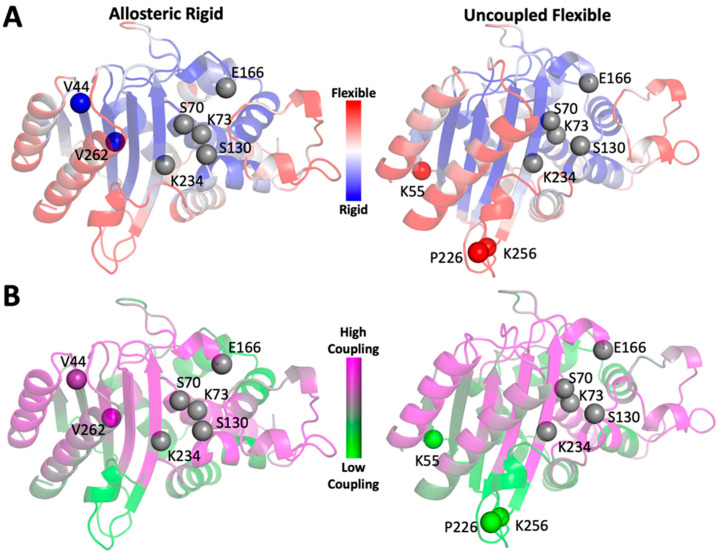
The dfi (**A**) and dci (**B**) values of each residue in TEM-1 are calculated and mapped onto the structure of TEM-1, which is shown as color coded cartoons. Catalytic residues are shown as grey spheres. Rigid and flexible residues used in this study are shown as spheres that are colored by either their dfi (**A**) or dci (**B**) score. Allosteric rigid residues, V44 and V262, have low dfi scores and high allosteric dynamic coupling with the active site residues. Residues K55, P226, and K256 are both highly flexible and exhibit low allosteric dynamic coupling to the active site.

We selected two residues in TEM-1 (V44 and V262) as targets for our study. Not only do both residues have low dfi scores (%dfi value < 0.2) (Figure 2A), but they are highly coupled to the active site (%dci > 0.7) (Figure 2B). These two residues were of particular interest to us because they are over 10 Å away from the active site and are located on adjacent β-strands with side chains facing opposite domains. We also identified three distal, flexible residues in TEM-1 (K55, P226, and K256) with high dfi scores (%dfi > 0.8) (Figure 2A) and low coupling to active site residues as evaluated by the dci metric (%dci < 0.4) (Figure 2B) and over 10 Å away from the active site to serve as controls. Alteration of the protein environments surrounding allosteric rigid residues would be expected to substantially modify protein function if our hypothesis is correct. Alternatively, modification of amino acids surrounding flexible residues with low dynamic coupling to the active site would be expected to result in proteins with native-like functions. All of the allosteric rigid and uncoupled flexible residues we targeted for design are over 10 Å from the nearest catalytic residue, which suggests that mutations in their vicinities should only have an indirect effect on the active site unless other factors (e.g., dynamic coupling) are at play.

### 2.2. Computational Design of TEM-1 Variants

In order to alter the amino acid compositions surrounding both the rigid and flexible residue positions, we used the Rosetta computational protein design suite [36]. The Rosetta software employs a Monte Carlo sampling protocol to randomize the identity and conformation (rotamer) of a randomly chosen residue; the fitness of the mutated protein is then assessed using the Rosetta energy function [37]. In the course of a single design trajectory, the Monte Carlo sampling algorithm is applied iteratively to a set of user-defined residues (see below). 

**Figure 3:**
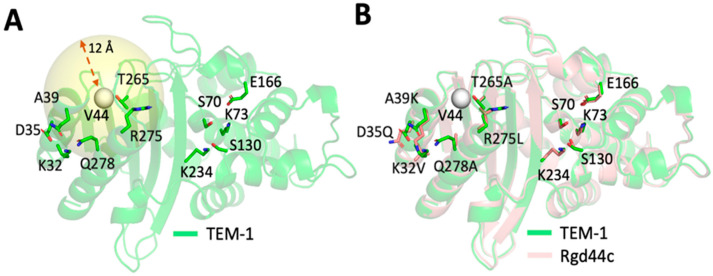
Our general computational protein design strategy is shown schematically using the designed protein Rgd44c as an example. (**A**) Residues within an 8–12 Å sphere surrounding a given residue (V44 in this example) are considered as candidates for mutation. (**B**) A combination of mutations surrounding the target residue are generated using the RosettaDesign algorithm and scored using the Rosetta energy function. An overlay of the Rgd44c design model with TEM-1 (**B**) indicates that this design protocol creates a diversity of mutations within the design sphere while leaving active site residues untouched. The target rigid residue (V44) is shown as a white sphere in both panels. Both catalytic and designed residues are shown as sticks.

We sought to develop a computational protocol within Rosetta that would substantially alter the chemical properties of the native amino acids without negatively affecting the protein’s ability to fold. To do this, the RosettaDesign algorithm [38] was used to randomly mutate residues within “design spheres” that had radii from 8–12 Å surrounding each of the target residues (Figure 3A). Slight alterations to the conformation of the peptide backbone were allowed only for residues that fell within the design sphere. A second shell was also defined that extended 4 Å beyond the inner design sphere. Residues in this shell were precluded from mutating but were energetically minimized in the context of adjacent, mutated residues. Independent design trajectories were carried out for all rigid and flexible residues. The two rigid (V44 and V262) and three flexible (K55, P226 and K256) residues that served as targets for our studies were also prohibited from mutating during the design calculations (Figure 3B). Finally, catalytic residues (S70, K73, S130, E166, K234) were also maintained as their native identities and conformations during the design process. The designed proteins contained between two and eleven mutations with an average of seven mutations per protein. Ultimately, 64 unique designed proteins were generated using this approach.

### 2.3. Selection of the Designed Proteins Using Flexibility Profiles

**Figure 4:**
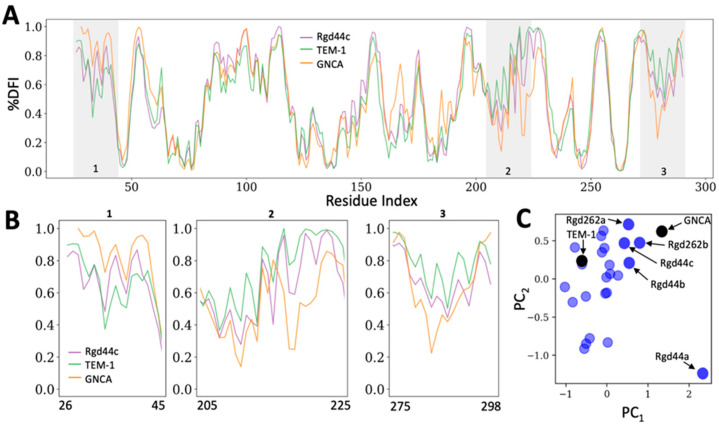
Dynamic analyses of TEM-1, GNCA, and the rigid designs. (**A**) Depiction of the dfi profile of TEM-1 (green), GNCA (orange) and variant Rgd44c (purple); Rdg44c is chosen as an example for illustrative purposes. (**B**) Portions of the full dfi profile of each protein (**A**) are expanded to highlight dynamic differences between the three proteins. A shift towards a GNCA-like dfi profile is an indication of a change in dynamical characteristics of a protein. (**C**) Principal Component Analysis (PCA) of the rigid designs. The first (x-axis) and second (y-axis) principal components have weights of 3.5 and 2.7, respectively. Designs chosen for experimental characterization are highlighted using darker colors and labeled with the design name.

To assess how the computationally designed mutations affected TEM-1 dynamics, we subjected all designed proteins to a 1 µs molecular dynamics (MD) simulation followed by analysis using the dfi metric (Figure 4A). In order to rapidly compare the dfi profiles of our designed proteins to those of TEM-1 and GNCA, we used a 2D principal component analysis (PCA). The PCAs both simplified our data and allowed for the facile visualization of relationships between the calculated dynamic profiles of the designed proteins (Figure 4B). PCAs generated from our rigid designs showed a diverse distribution in both the first and second principal components (Figure 4C). On the PCA, several designed proteins were positioned relatively closer to GNCA in both components. We chose a subset of five such designs in which the allosteric rigid residues had been targeted (henceforth referred to as “rigid designs”) for experimental characterization (Figure 4C). Four of the five rigid designs (Rdg44b, Rdg44c, Rdg262a, and Rdg262b, where the number in each name corresponds to the rigid residue that was targeted in the design calculations) clustered slightly away from TEM-1 and towards GNCA on both axes of the PCA; alternatively, Rdg44a, clustered near GNCA on the first principal axis but appeared as an outlier on the second axis. We hoped that experimental characterization of Rdg44a might help elucidate the parameters captured in each of the two principal components. It should be mentioned that only four among the five rigid designs that were chosen for characterization had Rosetta scores that were lower (lower Rosetta scores imply lower energies) than TEM-1. The Rosetta score of Rdg262a was higher than TEM-1, but we selected this design for experimental characterization due to the fact that it clustered near GNCA in both axes of the PCA.

To analyze the designed proteins in which flexible, uncoupled residues were targeted (henceforth referred to as “flexible designs”), we generated a PCA in which all flexible design candidates were compared to TEM-1, GNCA and all the rigid designs including those that were not selected for characterization (Supplementary Figure S1). Although a wide distribution of flexible designs was observed in this PCA, many of them clustered near TEM-1; a smaller subset clustered near the rigid designs we previously selected for characterization. In an effort to avoid biases that might have arisen if we chose only flexible designs that clustered with TEM-1 for analysis, we opted to experimentally characterize four flexible designs (Flx226a, Flx226b, Flx226c and Flx55) that clustered near the rigid designs chosen for experimental characterization and only one (Flx256) that clustered near TEM-1 (Supplementary Figure S1). Although clustering in similar locations in the PCA would suggest that the two proteins should have similar properties, it is difficult to infer what feature is represented on each axis of the PCA. We hoped that the diverse selection of proteins chosen for characterization would therefore provide information regarding whether rigid residues serve as hubs of dynamic control and also whether or not the PCA is a useful metric for discriminating between proteins with different activity and thermostabilities.

### 2.4. Experimental Analysis of the Designed Proteins

As GNCA and TEM-1 differ substantially with respect to thermostability (90.3 °C and 56.4 °C, respectively) and activity against penam β-lactam antibiotics (GNCA is ~2 orders of magnitude less efficient at degrading ampicillin than TEM-1), we chose to focus our analyses of the designed proteins on these characteristics. To do this, genes encoding each of the selected rigid and flexible designs were first cloned into the pET29b expression plasmid. Sequenced confirmed plasmids were transformed into a BL21 Star (DE3) *Escherichia coli* expression strain in preparation for further analyses.

We assessed the resistance of our designed proteins to penam β-lactams by establishing the minimal inhibitory concentration of ampicillin (MIC_amp_) for each of our designed proteins using the protocol of Wiegand et al. [39]. (See Materials and Methods for detailed protocols). Briefly, BL21 Star (DE3) cells harboring a pET29b plasmid that contained a gene encoding one of our variants were grown in a liquid medium containing a range of ampicillin concentrations and 1 mM isopropyl β-D-1-thiogalactopyranoside (IPTG), which induced overexpression of our TEM-1 variants. The ability of cells to grow at each ampicillin concentration was determined by measuring the optical density at 600 nm (O.D._600_); the lowest antibiotic concentration that inhibited growth was recorded. All rigid designs exhibited either minimal or no activity against ampicillin (Table 1). The two rigid designs that showed the highest activity against ampicillin, Rdg44c and Rdg262b, had MIC_amp_ values of 26 µg/mL, which is two orders of magnitude less efficient than wild type TEM-1 (MIC_amp_ = 1500 μg/mL), but is only half that of GNCA (MIC_amp_ = 43 μg/mL). Alternatively, the MIC_amp_ values of all the flexible designs were in the range of 375-1500 μg/mL (Table 1) which is on par with wild type TEM-1.

Two possible explanations for the lack of activity against ampicillin observed in our rigid designs are: (1) that only poor protein expression was achieved or (2) that they did not fold into native-like structures; neither of these possibilities are directly examined in MIC assays. We therefore expressed and purified each of the designed proteins and assessed their abilities to adopt native-like structures using circular dichroism (CD) spectroscopy. All designed proteins were observed to express solubly (Supplementary Figure S2). However, two of the rigid designs, Rdg44a and Rdg262a, had a high propensity to aggregate during the purification process, which precluded further characterization. In contrast, no aggregation of any of the flexible designed proteins was observed throughout the purification process. We subjected all purified proteins to both wavelength scans and thermal melts using CD (see Materials and Methods), which allowed determination of the melting temperature (T_m_) of each protein (Supplementary Figure S3). The T_m_s of all flexible designs fell into a range (53.2 °C to 58.5 °C) that was within ~3 °C of the T_m_ of TEM-1 (56.4 °C, Table 1). Alternatively, the T_m_s of the rigid designs varied greatly. Although the least stable of the allosteric rigid designs (Rdg262b) exhibited a T_m_ that was on par with TEM-1, two others exhibited marked increases in stability. Namely, Rdg44b and Rdg44c were measured to have T_m_s of 63.1 °C and 66.4 °C, respectively, which correspond to increases of ~6 °C and 10 °C relative to TEM-1.

The residues targeted for design in this study exhibit a broad distribution of distances from the active site. For example, the two rigid residues (V44 and V262) are closer to the active site than any flexible residues that were targeted for design with distances of 10.1 Å and 17.3 Å, respectively, while the distance of the flexible residues from a catalytic residue ranged from 17.5 Å–22.1 Å. We therefore sought to assess whether or not a correlation existed with respect to the distance from a targeted residue to the active site and altered enzymatic function. To do this, we calculated the distances between the Cαs of all residues mutated during the design process and the Cα of the nearest catalytic residue for all experimentally characterized proteins (Supplementary Table S1) using the PyMOL software (The PyMOL Molecular Graphics System, Version 4.3; Schrödinger, LLC: New York, NY, USA).

The two designed proteins that had the shortest distances between a mutated residue and one of the catalytic residues both targeted residue 262 (Rdg262a and b). Rdg262a carries a mutation at position 233, which is directly adjacent in sequence space to catalytic residue 234. Rdg262b contains the next shortest distance between a mutation and an active site residue at 5.8 Å. Rdg262a showed no activity against ampicillin; it is possible that the observed lack of activity is due to the protein’s instability and/or propensity to aggregate as observed during purification. Alternatively, Rdg262b possessed an identical T_m_ to TEM-1 but showed minimal activity against ampicillin despite containing a mutation that is only ~6 Å away from a catalytic residue. On the other end of the spectrum, the nearest mutations to any catalytic residue in two of the flexible designs, Flx226a and c, are 18.5 and 17.5 Å away, respectively. Both of these TEM-1 variants showed near native activity against ampicillin, which is consistent with the fact that mutations that are both distant from and uncoupled to the active site should have little effect on activity.

In the remaining designs, the distribution of distances between the nearest catalytic residue and a designed mutation are much more similar irrespective of whether rigid or flexible residues were targeted. For example, Rdg44a and Flx226b both have mutations that are 12.1 Å from a catalytic residue and Rdg44c and Flx55 have mutations that are 9.7 Å and 9.8 Å away from the catalytic residues, respectively. As these pairs of proteins contain one rigid and one flexible design and also exhibit similar distances between the nearest mutation and any catalytic residue, they appear to provide a direct test of the implications of targeting mutations to flexible vs. rigid residues. Interestingly, Rdg44a was highly unstable and aggregation prone despite only having mutations over 10 Å away from the catalytic residues. In contrast, Rdg44c had activity against ampicillin that was three orders of magnitude less than the wild type protein, but also showed a 10 °C increase in T_m_ relative to TEM-1. Alternatively, both flexible designs (Flx226b and Flx55) maintained substantial activity against ampicillin and exhibited T_m_s that were within 3 °C of wild type TEM-1 (Table 1). These data further support the notion that rigid, highly coupled residues play a large role in determining both the activity and physical properties of TEM-1. Furthermore, the fact that the rigid designs that adopted a native-like fold showed a substantial decrease in activity supports the notion that our dci metric can provide meaningful information regarding residues that may be able to affect protein function via allosteric dynamic coupling to the active site.

### 2.5. Dynamics Analysis of the Designed Proteins

**Figure 5:**
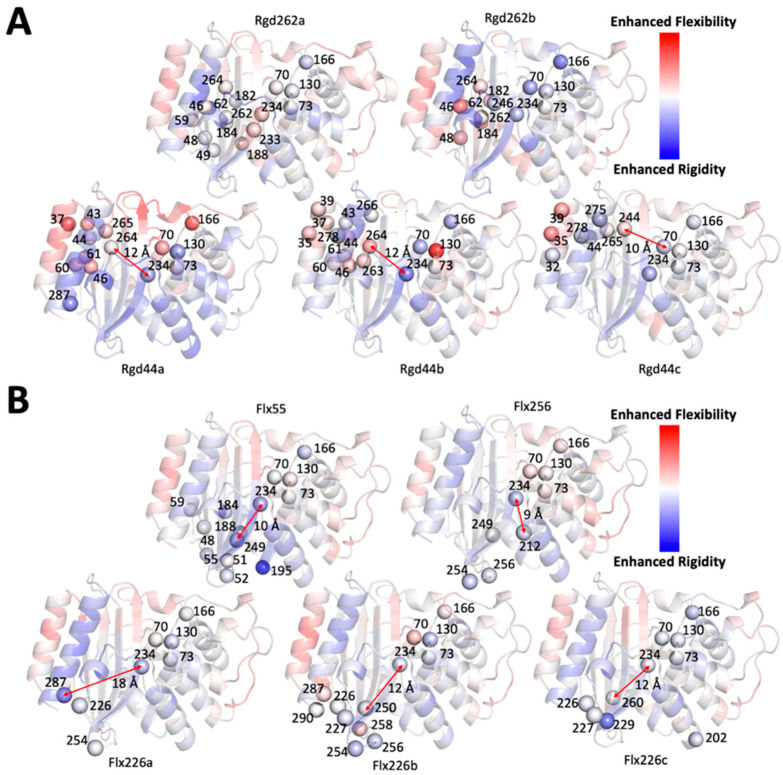
The change in the dynamics profiles of experimentally characterized rigid (**A**) and flexible (**B**) designs (∆dfi values) are mapped onto the TEM-1 structure. Point mutations around the residues targeted for design and the catalytic residues in TEM-1 are shown as spheres and labeled with their residue indices. The distance between the mutations closest to the catalytic residues are marked with red arrows and labeled with the corresponding distance in angstroms. The minimum distance in most designs is larger than 10 Å (Rgd262a and b and Flx256 are exceptions), which suggests that the changes in dynamics of catalytic residues is due to distal allosteric communication with the active site in many instances.

Experimental characterization of our designed proteins demonstrated that the MIC_amp_ values of the rigid designs were significantly reduced relative to both TEM-1 and the flexible designs irrespective of the distances between the nearest mutations and the catalytic residues. This suggests that changes in the local network of interactions surrounding rigid residues that exhibit long-range dynamic coupling with the active site may allosterically alter the flexibility of active site residues. In order to further analyze this possibility using our computational metrics, we calculated the flexibility of the active site residues in both sets of designed proteins using the dfi metric. The dfi values of each catalytic residue in our experimentally characterized proteins were subtracted from those of TEM-1 to generate a Δdfi profile (Supplementary Figure S4A). A clear difference between the Δdfi values of the catalytic residues of the rigid and flexible designs was observed (Figure 5). Namely, the catalytic residues in the rigid designs underwent a greater change in relative flexibility (both increases and decreases) compared to the flexible designs. Alternatively, the relative flexibilities of the catalytic residues in the flexible designs exhibited a narrower distribution centered at zero (Supplementary Figure S4B). These data support the notion that the rigid residues we chose are highly coupled to the active site (as suggested by our original dci analysis) and also that targeting the local interaction of allosteric rigid residues can indeed alter the flexibilities of residues, even if they are separated by substantial distances.

Our experimental results and the detailed dfi profiling of the experimentally characterized designs brought to light the fact that our initial PCA analysis did not appear to adequately discriminate between the activities of the designed proteins. Although designs in which rigid, coupled residues were targeted often possessed vastly different properties than those in which flexible, uncoupled residues were targeted, many of these designs clustered in similar areas of the PCA (Supplementary Figure S1). Therefore, we sought to develop a new metric that might have a greater discriminatory ability than the PCA alone. We developed an iterative method that we have termed the Dynamic Distance Analysis (dda) in which the “dynamic distance” of a designed protein to either TEM-1 or GNCA is computed relative to those of randomly selected groups of designed proteins. As the distance between any two proteins in a PCA (based on their three principal eigenvectors, see Methods and Materials) depends on the component proteins used to generate that PCA, randomly selected sets of designed proteins should yield a much better picture of the true relationship between a given designed protein and a target protein (TEM-1 and GNCA).

**Figure 6:**
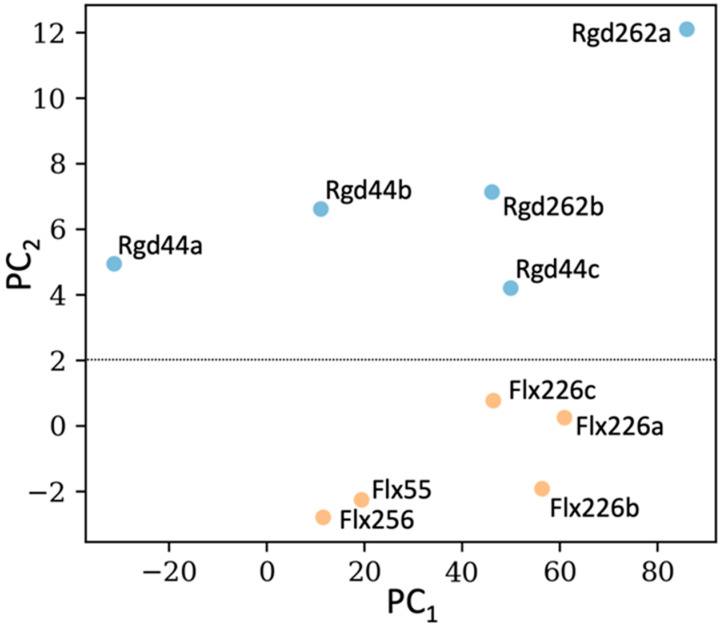
The dynamic distances are clustered for all characterized allosteric rigid (blue) and uncoupled flexible (orange) designs. The weights of PC_1_ and PC_2_ are 250 and 30, respectively. The rigid designs and the flexible designs cluster separately. Designed proteins with similar MIC_amp_ values, (Flx55 and Flx256), (Flx226c and Flx226a), (Rdg262b and Rdg44c) cluster in the same vicinity.

To generate the dda profiles of our designed proteins, we used a bootstrapping approach in which we first generated multiple PCAs using small, randomly chosen subsets of designed proteins and then iteratively measured the distances between the dfi profiles of each designed protein and both GNCA and TEM-1. (Supplementary Figure S5) When we clustered the dda profiles of the rigid and flexible designs using a new PCA; a clear separation between the two emerges (Figure 6), which correlates well with their biophysical characterization. For example, flexible designs Flx55 and Flx256 cluster together in our dda analysis and also possess similar MIC_amp_ values (750 µg/mL). Similarly, Flx226a and Flx226c, whose MIC_amp_ values are the same as TEM-1 (1500 µg/mL), also appear in very similar regions of the dda PCA. The two rigid designs, Rgd44a and Rgd262a, which exhibited aggregation during purification, are both found as outliers in the dda clustering. Notably, Rgd44c and Rgd262b, which exhibit higher thermostabilities and similar MIC_amp_ values to TEM-1, are also clustered in the same vicinity.

In an effort to assess whether or not the trends observed in the dda analyses of experimentally characterized proteins were universal, we applied dda to all the designed proteins, even those not chosen for characterization. Interestingly, the dynamic distances of the rigid designs are biased away from TEM-1 relative to their flexible design counterparts (Supplementary Figure S6A); conversely, the flexible designs form a narrower distribution that is closer to TEM-1. This suggests that flexible residues that are not coupled to the active site do not likely contribute to the collective motion of the protein as substantially as do rigid residues. When the distances of our designed proteins to GNCA are considered, the uncoupled flexible designs display a sharp, narrow distribution that is distant from GNCA (Supplementary Figure S6B). Alternatively, the distribution of the rigid designs is broad and contains proteins with dynamic profiles that are more like that of GNCA. These data suggest that the re-design of the environment surrounding rigid residues appears to alter the dynamics of TEM-1 more substantially than when the environment surrounding uncoupled flexible residues is targeted.

## 3. Conclusions

The goal of this work was to better understand the relationship between structure and function in the TEM family of β-lactamases. Building on previous evolutionary studies on the β-lactamase enzyme TEM-1 [15], we explored the hypothesis that rigid residues can serve to both establish the global dynamic profile of the enzyme and exert substantial influence over physical properties (e.g., substrate specificities) so long as long-range coupling exists between the rigid residues and the active site. To explore this, we used the Rosetta computational protein design software to re-design the local network of interactions surrounding residues that fit the aforementioned criteria. Our designed proteins were analyzed using computational metrics that assessed both the global dynamic profile and the allosteric coupling of each residue to the active site. Based on these metrics, a subset of our designed proteins was selected for experimental characterization.

Ten designed TEM-1 variants were characterized with respect to the minimal inhibitory concentration of ampicillin as well as thermostability. These data suggested that targeting mutations to environments surrounding rigid residues that were highly coupled to the active site often resulted in a substantial shift in protein stability and function; alternatively, targeting flexible, uncoupled residues resulted in protein variants with more native-like activities and thermostabilities. Namely, when mutations were targeted to the vicinity of two rigid residues that do not directly interact with the active site, but which are highly coupled to it, a substantial reduction in TEM-1′s ability to degrade its native substrate was observed in all cases even though native-like folds were maintained in many cases. Alternatively, thermostabilities and activities against TEM-1′s native substrate were maintained in a set of designed proteins in which residues that were neither rigid nor predicted to be coupled to the active site were targeted for mutagenesis. These results are consistent with our computational analyses of the designed proteins’ dynamics. Namely, it appears that altering the local interactions surrounding rigid residues that are highly coupled to the active site can allosterically alter the flexibility profiles of active site residues at a distance, which can in turn alter the biophysical properties of the enzyme. In an effort to identify an analytical method that was more informative as to the activities that designed proteins might possess, we developed a novel metric that measures the “dynamic distance” between two proteins. Many of our designed proteins with similar functional properties were observed to cluster together when analyzed by this algorithm. These results not only further support the potential importance of mutations in the vicinity of rigid residues, but also support the fact that coupling between distal residues and the active site can have profound effects on enzyme activities.

The relationship between protein dynamics and function is highly complex and studying it represents an exceedingly difficult challenge [8,9,24,40,41,42]. Our approach represents a new method for exploring this subject in a highly directed manner. We hope that additional application of these methods to distinct residues in TEM-1 will ultimately provide a more complete understanding of the complex dynamic landscape present in this class of proteins. This could not only facilitate a rapid prediction of the biochemical properties of new clinical isolates but could also pave the way for the development of new antibiotics that specifically target new protein conformations accessible only through alterations of the global dynamic profile. Finally, the methods reported here could also find use in understanding the dynamic profiles of other enzyme classes, which could have profound implications from the perspective of understanding and treating diseases.

## 4. Materials and Methods 

### 4.1. Molecular Dynamics (MD)

The AMBER software package was utilized for simulating all β-lactamases in this study. Each system was parameterized with the ff14SB force field and the explicit water model TIP3P [43,44]. The solvation box was assigned as 16 Å. The system was neutralized by sodium and chloride ions and minimized for 11,000 steps using the steepest descent algorithm. Isothermal, isobaric, and constant number of particles ensemble production trajectories were performed at 300K and 1 bar pressure. For each production, a 1 µs simulation was conducted. The residue covariances were calculated using a 50 ns length window shifted by 10 ns (example: 1–50 ns, 10–60 ns, etc.) over the course of the trajectories.

### 4.2. Dynamic Flexibility Index (dfi)

The dfi metric [16,19,21] calculates the relative flexibility/rigidity of a residue in a protein by incorporating the residue covariances. The protein can be modeled with the Elastic Network Model (ENM) in which harmonic springs connect Cαs [35]. Taking the second derivatives of the potential forms a Hessian matrix, *H* Equation (1). The inverse of the Hessian matrix is proportional to the covariance matrix. The models based on ENM cannot capture changes in the dynamics of the designed variants based on Cα positions alone. Therefore, we substituted the inverse of the Hessian with the covariance matrices from MD trajectories to capture the effect of mutations on the protein conformations. The covariance matrix, *G*, contains the residue covariances, obtained by the MD trajectories Equations (2) and (3) [17,19,28,29,45].

(1) $${\left[\Delta R\right]}_{3Nx1}={\left[H\right]}_{3Nx3N}^{-1}{\left[F\right]}_{3Nx1}$$

(2) $${\left[\Delta R\right]}_{3Nx1}={\left[G\right]}_{3Nx3N}{\left[F\right]}_{3Nx1}$$

(3) $$dfi_{i}=\frac{\sum_{j=1}^{N}{\left|\Delta R^{j}\right|}_{i}}{\sum_{i=1}^{N}\sum_{j=1}^{N}{\left|\Delta R^{j}\right|}_{i}}$$

The residue response vector (Δ*R*) is the resultant vector containing the fluctuation responses from multiplying the covariance matrix with the force vector, F. ${\left|\Delta R^{j}\right|}_{i}$ denotes the magnitude of the residue response fluctuation vector of position i, when j is exposed to a random force vector.

### 4.3. Dynamic Coupling Index (dci) 

The dynamic coupling index (dci) [17,19,45] measures the degree of dynamic coupling between two residues. Namely, it captures the strength of displacement of a residue *i* upon perturbation of a distinct residue *j*, relative to the average fluctuation response of position *i* when all of the positions within a structure are perturbed. Generally, this metric is used to establish the communication between a functionally important residue and other residues within the protein that are many angstroms away. The dynamic coupling index of a given residue i is calculated using the equation below Equation (4):

(4) $$dci_{i}=\frac{\sum_{j}^{N_{Functional}}{\left|\Delta R^{j}\right|}_{i}/N_{Functional}}{\sum_{j=1}^{N}{\left|\Delta R^{j}\right|}_{i}/N}$$

where ${\left|\Delta R^{j}\right|}_{i}$ corresponds to the magnitude of the residue response vector (Δ*R*) for residue *i* when residue *j* is perturbed. The dci score thus provides information on the allosteric behavior of a location associated with active site dynamics. A high dci value implies strong coupling between active sites, inversely, a low scoring position is regarded as weakly coupled to the active site [17,19,45].

### 4.4. Dynamic Distance Calculation 

Principal Component Analysis (PCA) was used to compare and cluster the flexibility profiles of the designed TEM-1 variants with respect to TEM-1 and GNCA. However, because the output of a PCA is dependent on the input data, the calculated distances between any designed protein and TEM-1 or GNCA can change with the inclusion of new or distinct data points (e.g., a different set of designed proteins). To account for this, we employed an iterative, random sampling approach to capture the relative distance of a designed protein from TEM-1 and from GNCA (Supplementary Figure S5).

For every designed TEM-1 variant, a dataset containing the target design, TEM-1, GNCA and an additional seven randomly chosen designs was constructed and used to generate a PCA. Namely, the dfi profiles of these ten proteins were merged into a matrix, X, of dimension Equation (5):

(5) (m × n)

Here, *m* is the total number of datasets that are clustered together, which each have *n* number of attributes (*n* = total number of residues). Singular value decomposition of X was then carried out as follows Equation (6):

(6) $${\left[X\right]}_{m\times n}={\left[U\right]}_{m\times m}{\left[\Sigma \right]}_{m\times n}{\left[V\right]}_{n\times n}$$

Here, *U* and *V* are unitary matrices with orthonormal columns and are called left singular vectors and right singular vectors, respectively, and Σ is a diagonal matrix with diagonal elements known as singular values of *X*.

The singular values of *X*, by convention, were arranged in a decreasing order of their magnitude, *σ* = {*σ_i_*} representing the variances in the corresponding left and right singular vectors. The set of the highest singular values (representing the largest variance in the orthonormal singular vectors) can be interpreted to show the characteristics in the data *X* and the right singular vectors create orthonormal basis which spans the vector space representing the data. The left singular vectors contain weights indicating the significance of each attribute in the dataset as Equation (7):

(7) $$w_{i}=\overset{r}{\underset{k=1}{\sum }}\sigma_{k}\left|u_{ik}\right|$$

Using these features of the decomposed singular vectors, we created another matrix, X* using only the highest three singular values which mimics the basic characteristics of the original dataset. It can be represented as Equation (8):

(8) $${\left[X^{*}\right]}_{mxr}={\left[V^{*}\right]}_{mxr}{\left[\Sigma^{*}\right]}_{rxr}$$

Here, Σ* contains only the largest 3 singular values and V* contains the corresponding right singular vectors. The data were then clustered hierarchically based on the pairwise distance between different proteins in the reconstructed dfi data with reduced dimensions. The distance between designed protein, *j*_1,_ and TEM-1, *j*_2_, was computed in the reduced dimension using three principal components Equation (9):

(9) $$d_{12}=\sqrt{\overset{3}{\underset{i=1}{\sum }}{\left(X_{i}^{*j_{1}}-X_{i}^{*j_{2}}\right)}^{2}}$$

We also calculated the distance between each designed TEM-1 variant and GNCA to measure the similarity in their flexibility profiles. The random selection of dataset was repeated a thousand times to create a diverse distance distribution and we called this distance profile analysis dynamic distance analysis (dda). The distributions were fit to a Gaussian mixture model with a Dirichlet prior to estimate the density and the mean of the dynamic distances [46]. The distributions and the mean distances were utilized for selecting the designed proteins that cluster close to GNCA and far from TEM-1 (Supplementary Figure S5).

### 4.5. Rosetta Design Protocol

A high-resolution (1.8 Å) structure of TEM-1 (PDB ID: 1btl) was processed to remove waters, non-proteinogenic molecules and a second copy of the protein in the asymmetric unit. The resulting structure was subjected to an energy minimization using the Rosetta relax protocol; detailed descriptions of all computational protocols used in this study can be found in the supplementary information section.

The relaxed 1btl structure was used as an input to the DesignAround protocol within Rosetta using the ref15 score function. This algorithm first identifies spheres with user-defined radii around a defined residue. Residues within these “design spheres” were subjected to in silico mutagenesis, conformational sampling and backbone minimization. 

### 4.6. Protein Expression and Purification

A pET24b plasmid encoding the gene for GNCA was a generous gift from Professor Jose Sanchez-Ruiz (Universidad de Granada). Genes encoding rigid design variants were codon-optimized for expression in *E. coli* cells. The native TEM-1 N-terminal periplasmic localization signal peptide (MSIQHFRVALIPFFAAFCLPVFA) was appended to the beginning of each gene; to facilitate purification, a C-terminal 6xHis affinity tag was added to the end of each gene. Genes encoding each rigid design were synthesized by IDT (Coralville, IA). The gene for wildtype TEM-1 was amplified from a pET21b vector using PCR. Genes encoding the rigid designs and TEM-1 were subcloned into the pET29b vector using the Gibson Assembly [47] at a site that placed them under the control of the T7lac promoter. Genes encoding the uncoupled flexible residue variants were synthesized and cloned into pET29b vectors by GenScript (Piscataway, NJ, USA).

The sequences of all plasmids containing TEM-1, GNCA, rigid or flexible designs were confirmed by Sanger sequencing and were transformed via electroporation into BL21 Star (DE3) *E. coli* cells. Cells containing plasmids encoding GNCA were grown in lysogeny broth (LB) at 37 °C with shaking at 250 rpm until an O.D._600_ of ~0.8 was reached. Isopropyl β-D-1-thiogalactopyranoside (IPTG) was then added to a final concentration of 1 mM to induce expression; cells were grown for 3 h post induction. Cells containing plasmids encoding TEM-1 were grown in LB media at 20 °C with shaking at 220 rpm until an O.D._600_ of ~0.8 was reached. Induction was again carried out with 1 mM IPTG and was allowed to proceed for 8–12 h. Cells containing plasmids encoding the rigid and flexible design variants were grown in 2xYT media to confluence overnight and pelleted by centrifugation. After resuspension in fresh 2xYT media, protein expression was induced with 1 mM IPTG and cells were grown for an additional 20 h at 20 °C with shaking at 220 rpm.

After expression, the cells were pelleted via centrifugation at 4100× *g* for 15 min and the media was discarded. The cells were resuspended in TBS (50 mM Tris pH 8.0, 500 mM NaCl) and were again centrifuged at 4100× *g* for 15 min; the supernatant was discarded. The pellet was incubated at room temperature for 15 min with SET buffer (20% sucrose, 1 mM ethylenediaminetetraacetic acid (EDTA), 30 mM Tris pH 8.0, 1 μM phenylmethylsulfonyl fluoride (PMSF), 1 mg/mL lysozyme). After centrifugation at 4100× *g* for 15 min, the supernatant was decanted and saved. The cells were then shocked to release the periplasmic contents with ice cold 100 mM MgCl_2_ at a 1:15 ratio of cell pellet weight to solution volume. Cells were vigorously agitated on ice for 15–30 min then centrifuged with the saved soluble fraction from the first stage at 4 °C for 60 min at 12,000× *g*.

The supernatant was then loaded onto a 5 mL nitrilotriacetic acid agarose (Ni-NTA) (Millipore Sigma, Burlington, MA, USA) column, washed with 5 column volumes of a low imidazole buffer (25 mM Tris pH 8.0, 150 mM NaCl, 15 mM imidazole), and eluted with a high imidazole buffer (25 mM Tris pH 8.0, 150 mM NaCl, 500 mM imidazole). All proteins were then subjected to a second purification step using anion exchange chromatography: Proteins were concentrated to a volume of 0.5–1 mL, diluted into the loading buffer (50 mM Tris, pH 9.0, 50 mM NaCl) and loaded directly onto the 5 mL Hi Trap Q Fast Flow column (Millipore Sigma, Burlington, MA, USA). The column was washed with 5 column volumes of the loading buffer and eluted with 50 mM Tris, pH 9.0 250 mM NaCl. Protein purity was verified by SDS-PAGE (Supplementary Figure S2).

### 4.7. Circular Dichroism Characterization of Protein Folding and Stability

Far-ultraviolet circular dichroism (CD) measurements were performed in triplicate on a Jasco J-815 spectrophotometer (Jasco, Inc, Easton, MD, USA) equipped with a Peltier temperature controller. Wavelength scans were measured from 300 to 180 nm at room temperature with 1 nm steps using a 1 nm bandwidth, 5 nm/min scan rate; reported data represent an average of three independent scans. Thermal melts were monitored by the absorption signal at 222 nm with a temperature slope of 5 °C/min. For wavelength scans and thermal melts, the purified protein was in a TBS buffer (10mM Tris 50 mM NaCl, pH 7.0) in a cuvette with a 1 mm pathlength. Protein concentrations were calculated in triplicate using the absorbance at 280 nm and absorption coefficients as calculated by the ProtParam tool in the Expasy software suite [48]. Protein concentrations ranged between 0.18–0.25 mg/mL for all scans. Thermal melt curves were fitted using nonlinear regression least squares fit with the Hill equation in the GraphPad Prism version 9.0.0 for Windows, GraphPad software, San Diego, California, USA.

### 4.8. MIC Assays

Minimal inhibitory concentrations of ampicillin (MIC_amp_) were performed in triplicate on 96-well plates [39]. For each designed protein, TEM-1 and GNCA, five colonies were picked from a fresh agar plate and used to inoculate a 5 mL culture of LB, which was grown to confluence overnight at 37 °C. Overnight cultures were diluted in LB with 1 mM IPTG to a final working concentration of 5 × 10^5^ cfu/mL. Three stock solutions of ampicillin were independently prepared at 6000 µg/mL in LB with 1 mM IPTG and each solution was subsequently diluted in steps of 0.5 through the addition of LB with 1 mM IPTG to yield a final range of concentrations of 6–3000 µg/mL. The ampicillin concentrations for GNCA and the rigid designs were prepared at 400 µg/mL in LB with 1 mM IPTG and each solution was diluted in steps of 0.6 for a final concentration range of 2–200 µg/mL. The 96-well plates were covered with a fitted lid and incubated at 37 °C for 20 h. All optical density measurements were carried out at 600 nm using a SpectraMax M5 (Molecular Devices, LLC, San Jose, CA, USA); the absorbance of the buffer was subtracted from each measurement. To establish the lowest concentration of antibiotic that inhibited growth, a buffer-subtracted value ≥ 0.1 was used as the threshold for bacterial growth in each well. The MIC_amp_ was determined to be the lowest concentration of ampicillin that inhibited growth of the *E. coli* cells.

## Figures and Tables

**Table 1:** Minimal Inhibitory Concentrations (MIC_amp_) and melting temperatures of the TEM-1 variants.

Variant	Minimal Inhibitory Concentration of Ampicillin MIC_amp_ (µg/mL)	Melting Temperature T_m_ (°C)
GNCA	43	90.3
TEM-1	1500	56.4
Rdg44a	<2 **	NM
Rdg44b	<2	63.1
Rdg44c	26	66.4
Rdg262a	<2 **	NM
Rdg262b	26	56.4
Flx226a	1500	57.4
Flx226b	375	53.2
Flx226c	1500	55.6
Flx256	750	58.1
Flx55	750	58.5

Minimal Inhibitory Concentrations for ampicillin (MIC_amp_) values were determined in lysogeny broth. Melting temperatures (T_m_) were determined using circular dichroism. NM indicates that a T_m_ was not established for this protein due to aggregation during purification. ** Because these variants precipitated out of solution during purification, it is difficult to know whether these values accurately reflect their activities *in cellulo*.

## Data Availability

Relevant data and the code for the dfi and dci analyses have been deposited at the following github address: https://github.com/SBOZKAN/DFI-DCI (accessed on 11 March 2021).

## Supplementary Materials

The following are available online at https://www.mdpi.com/1422-0067/22/6/2895/s1, Supplementary Figures S1–S6, Supplementary Table S1, detailed Rosetta methods.

## References

[1] Coulson A. (1985). ß-Lactamases: Molecular Studies. Biotechnol. Genet. Eng. Rev..

[2] Bush K. (2018). Past and present perspectives on β-lactamases. Antimicrob. Agents Chemother..

[3] Brandt C., Braun S.D., Stein C., Slickers P., Ehricht R., Pletz M.W., Makarewicz O. (2017). In silico serine β-lactamases analysis reveals a huge potential resistome in environmental and pathogenic species. Sci. Rep..

[4] Gobeil S.M.C., Ebert M.C.C.J.C., Park J., Gagné D., Doucet N., Berghuis A.M., Pleiss J., Pelletier J.N. (2019). The Structural Dynamics of Engineered β-Lactamases Vary Broadly on Three Timescales yet Sustain Native Function. Sci. Rep..

[5] Brown C.A., Hu L., Sun Z., Patel M.P., Singh S., Porter J.R., Sankaran B., Prasad B.V.V., Bowman G.R., Palzkill T. (2020). Antagonism between substitutions in β-lactamase explains a path not taken in the evolution of bacterial drug resistance. J. Biol. Chem..

[6] Cortina G.A., Kasson P.M. (2018). Predicting allostery and microbial drug resistance with molecular simulations. Curr. Opin. Struct. Biol..

[7] Cortina G.A., Hays J.M., Kasson P.M. (2018). Conformational Intermediate That Controls KPC-2 Catalysis and Beta-Lactam Drug Resistance. ACS Catal..

[8] Singh M.K., Dominy B.N. (2012). The Evolution of Cefotaximase Activity in the TEM β-Lactamase. J. Mol. Biol..

[9] Orencia M.C., Yoon J.S., Ness J.E., Stemmer W.P., Stevens R.C. (2001). Predicting the emergence of antibiotic resistance by directed evolution and structural analysis. Nat. Genet..

[10] Wang X., Minasov G., Shoichet B.K. (2002). Evolution of an Antibiotic Resistance Enzyme Constrained by Stability and Activity Trade-offs. J. Mol. Biol..

[11] Fair R.J., Tor Y. (2014). Antibiotics and Bacterial Resistance in the 21st Century. Perspect. Med. Chem..

[12] Doucet N., Savard P.-Y., Pelletier J.N., Gagné S.M. (2007). NMR Investigation of Tyr105 Mutants in TEM-1 β-Lactamase. J. Biol. Chem..

[13] Kim H., Zou T., Modi C., Dörner K., Grunkemeyer T.J., Chen L., Fromme R., Matz M.V., Ozkan S.B., Wachter R.M. (2015). A Hinge Migration Mechanism Unlocks the Evolution of Green-to-Red Photoconversion in GFP-like Proteins. Structure.

[14] Modi T., Huihui J., Ghosh K., Ozkan S.B. (2018). Ancient thioredoxins evolved to modern-day stability–function requirement by altering native state ensemble. Philos. Trans. R. Soc. B: Biol. Sci..

[15] Zou T., Risso V.A., Gavira J.A., Sanchez-Ruiz J.M., Ozkan S.B. (2014). Evolution of Conformational Dynamics Determines the Conversion of a Promiscuous Generalist into a Specialist Enzyme. Mol. Biol. Evol..

[16] Gerek Z.N., Ozkan S.B. (2011). Change in Allosteric Network Affects Binding Affinities of PDZ Domains: Analysis through Perturbation Response Scanning. PLoS Comput. Biol..

[17] Larrimore K.E., Kazan I.C., Kannan L., Kendle R.P., Jamal T., Barcus M., Bolia A., Brimijoin S., Zhan C.-G., Ozkan S.B. (2017). Plant-expressed cocaine hydrolase variants of butyrylcholinesterase exhibit altered allosteric effects of cholinesterase activity and increased inhibitor sensitivity. Sci. Rep..

[18] Gerek Z.N., Keskin O., Ozkan S.B. (2009). Identification of specificity and promiscuity of PDZ domain interactions through their dynamic behavior. Proteins: Struct. Funct. Bioinform..

[19] Modi T., Ozkan S.B. (2018). Mutations Utilize Dynamic Allostery to Confer Resistance in TEM-1 β-lactamase. Int. J. Mol. Sci..

[20] Campitelli P., Modi T., Kumar S., Ozkan S.B. (2020). The Role of Conformational Dynamics and Allostery in Modulating Protein Evolution. Annu. Rev. Biophys..

[21] Kumar A., Butler B.M., Kumar S., Ozkan S.B. (2015). Integration of structural dynamics and molecular evolution via protein interaction networks: A new era in genomic medicine. Curr. Opin. Struct. Biol..

[22] Campitelli P., Guo J., Zhou H.-X., Ozkan S.B. (2018). Hinge-Shift Mechanism Modulates Allosteric Regulations in Human Pin1. J. Phys. Chem. B.

[23] Risso V.A., Gavira J.A., Mejia-Carmona D.F., Gaucher E.A., Sanchez-Ruiz J.M. (2013). Hyperstability and Substrate Promiscuity in Laboratory Resurrections of Precambrian β-Lactamases. J. Am. Chem. Soc..

[24] Salverda M.L., De Visser J.A.G., Barlow M. (2010). Natural evolution of TEM-1 β-lactamase: Experimental reconstruction and clinical relevance. FEMS Microbiol. Rev..

[25] Stiffler M.A., Hekstra D.R., Ranganathan R. (2015). Evolvability as a Function of Purifying Selection in TEM-1 β-Lactamase. Cell.

[26] Li Z., Bolia A., Maxwell J.D., Bobkov A.A., Ghirlanda G., Ozkan S.B., Margulis C.J. (2015). A Rigid Hinge Region Is Necessary for High-Affinity Binding of Dimannose to Cyanovirin and Associated Constructs. Biochemistry.

[27] Modi T., Campitelli P., Kazan I.C., Ozkan S.B. (2021). Protein folding stability and binding interactions through the lens of evolution: A dynamical perspective. Curr. Opin. Struct. Biol..

[28] Gerek Z.N., Kumar S., Ozkan S.B. (2013). Structural dynamics flexibility informs function and evolution at a proteome scale. Evol. Appl..

[29] Kumar A., Glembo T.J., Ozkan S.B. (2015). The Role of Conformational Dynamics and Allostery in the Disease Development of Human Ferritin. Biophys. J..

[30] NCBI BioProject Database. https://www.ncbi.nlm.nih.gov/bioproject/.

[31] Ambler R.P., Coulson A.F.W., Frère J.M., Ghuysen J.M., Joris B., Forsman M., Levesque R.C., Tiraby G., Waley S.G. (1991). A standard numbering scheme for the class A β-lactamases. Biochem. J..

[32] Jelsch C., Mourey L., Masson J.M., Samama J.P. (1993). Crystal Structure of Escherichia Coli TEM1 Beta-Lactamase at 1.8 A Resolution. Proteins.

[33] Risso V.A., Gavira J.A., Gaucher E.A., Sanchez-Ruiz J.M. (2014). Phenotypic comparisons of consensus variants versus laboratory resurrections of Precambrian proteins. Proteins Struct. Funct. Bioinform..

[34] Butler B.M., Gerek Z.N., Kumar S., Ozkan S.B. (2014). Conformational dynamics of nonsynonymous variants at protein interfaces reveals disease association. Proteins Struct. Funct. Bioinform..

[35] Atilgan C., Gerek Z., Ozkan S., Atilgan A. (2010). Manipulation of Conformational Change in Proteins by Single-Residue Perturbations. Biophys. J..

[36] Leaver-Fay A., Tyka M.D., Lewis S.M., Lange O.F., Thompson J., Jacak R., Kaufman K.W., Renfrew P.D., Smith C.A., Sheffler W. (2011). Rosetta3. Methods Enzymol..

[37] Alford R.F., Leaver-Fay A., Jeliazkov J.R., O’Meara M.J., DiMaio F.P., Park H., Shapovalov M.V., Renfrew P.D., Mulligan V.K., Kappel K. (2017). The Rosetta All-Atom Energy Function for Macromolecular Modeling and Design. J. Chem. Theory Comput..

[38] Kuhlman B., Dantas G., Ireton G.C., Varani G., Stoddard B.L., Baker D. (2003). Design of a Novel Globular Protein Fold with Atomic-Level Accuracy. Science.

[39] Wiegand I., Hilpert K., Hancock R.E.W. (2008). Agar and broth dilution methods to determine the minimal inhibitory concentration (MIC) of antimicrobial substances. Nat. Protoc..

[40] Knies J.L., Cai F., Weinreich D.M. (2017). Enzyme Efficiency but Not Thermostability Drives Cefotaxime Resistance Evolution in TEM-1 β-Lactamase. Mol. Biol. Evol..

[41] Zhang Y., Doruker P., Kaynak B., Zhang S., Krieger J., Li H., Bahar I. (2020). Intrinsic dynamics is evolutionarily optimized to enable allosteric behavior. Curr. Opin. Struct. Biol..

[42] Ma B., Tsai C.-J., Haliloğlu T., Nussinov R. (2011). Dynamic Allostery: Linkers Are Not Merely Flexible. Structure.

[43] Maier J.A., Martinez C., Kasavajhala K., Wickstrom L., Hauser K.E., Simmerling C. (2015). ff14SB: Improving the Accuracy of Protein Side Chain and Backbone Parameters from ff99SB. J. Chem. Theory Comput..

[44] Salomon-Ferrer R., Götz A.W., Poole D., Le Grand S., Walker R.C. (2013). Routine Microsecond Molecular Dynamics Simulations with AMBER on GPUs. 2. Explicit Solvent Particle Mesh Ewald. J. Chem. Theory Comput..

[45] Campitelli P., Ozkan S.B., Swint-Kruse L. (2020). Asymmetry in Dynamic Allosteric Residue Coupling (DARC) Interactions Captures Evolutionary Landscape. Biophys. J..

[46] Bishop C. (2006). Pattern Recognition and Machine Learning.

[47] Gibson D.G., Young L., Chuang R.-Y., Venter J.C., Hutchison C.A., Smith H.O. (2009). Enzymatic assembly of DNA molecules up to several hundred kilobases. Nat. Methods.

[48] Gasteiger E., Hoogland C., Gattiker A., Wilkins M.R., Appel R.D., Bairoch A., Walker J.M. (2005). Protein Identification and Analysis Tools on the ExPASy Server. The Proteomic Protocols Handbook.

