# Supplementary material for: The Role of Rigid Residues in Modulating TEM-1 β-Lactamase Function and Thermostability
Source: Int J Mol Sci. 2021 Mar 12;22(6):2895. doi: 10.3390/ijms22062895 (PMC7999226; doi:10.3390/ijms22062895)
Supplement: Supplementary file 1 [file ijms-22-02895-s001.pdf]

The Role of Rigid Residues in Modulating TEM-1 Function and Thermostability

Bethany Kolbaba-Kartchner, I. Can Kazan, Jeremy H. Mills, and S. Banu Ozkan

Supplementary Information

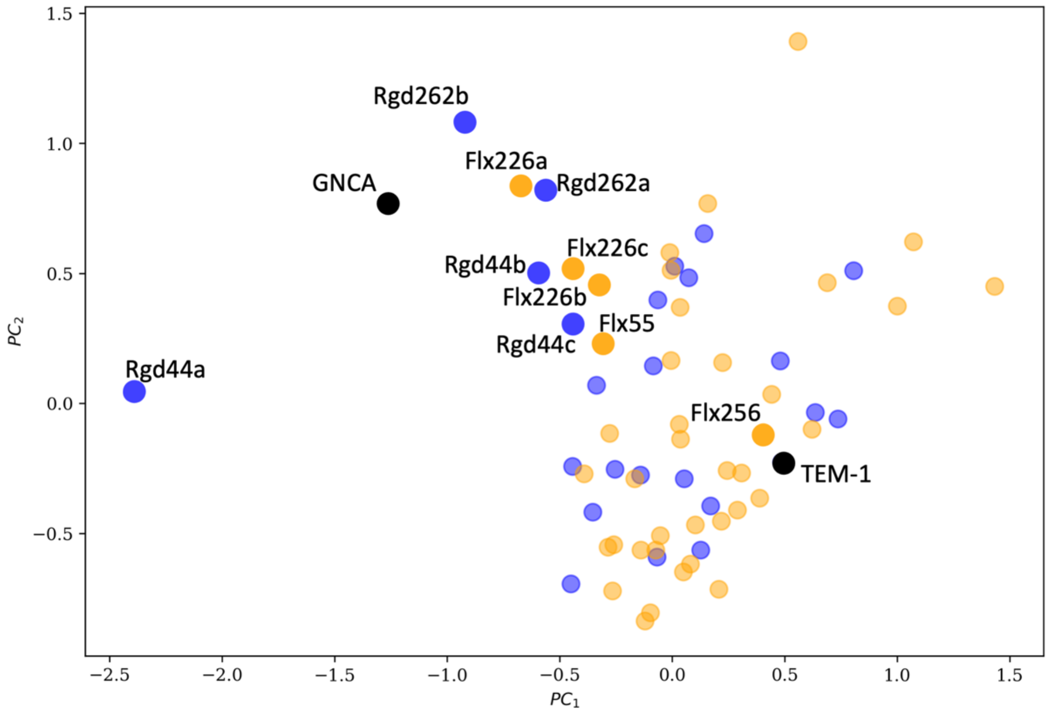

Supplementary Figure 1. PCA of a selection of the flexible and rigid designed proteins. The rigid designs with allosteric dynamic coupling to the active site are marked with blue dots. Uncoupled flexible designs are marked with orange dots. TEM-1 and GNCA are shown as black dots. For both rigid and flexible designs, the variants chosen for experimental characterization are named and highlighted with darker colors.

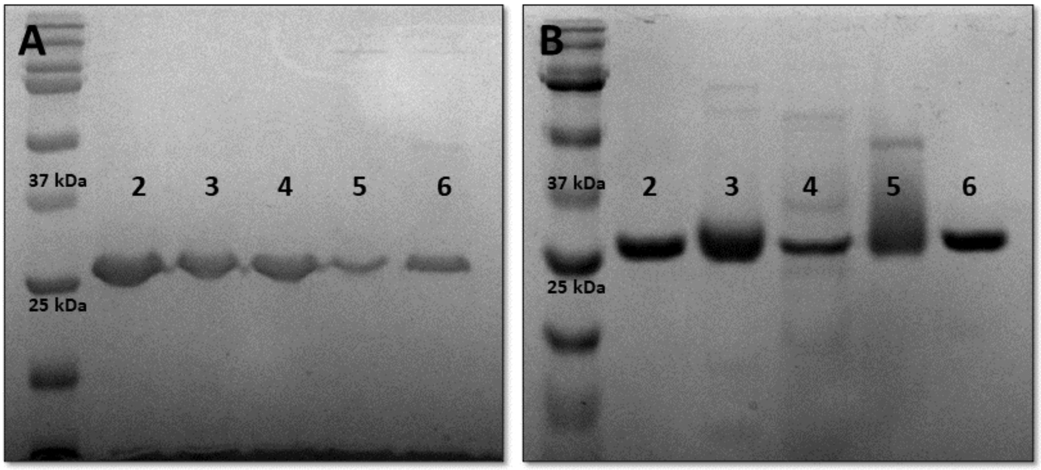

Supplementary Figure 2. 12% SDS PAGE gels of the purified designed proteins. The gels were stained with Coomassie Brilliant Blue G-250. For the gels, proteins were heat denatured. The protein standard (lane 1) is Bio-Rad Precision Plus Protein Kaleidoscope Prestained Protein Standards (A) Flx226a (lane 2) Flx226b (lane 3) Flx226c (lane 4) Flx256 (lane 5) Flx55 (lane 6) (B) TEM-1 (lane 2) GNCA (lane 3) Rdg44b (lane 4) Rdg44c (lane 5) Rdg262b (lane 6).

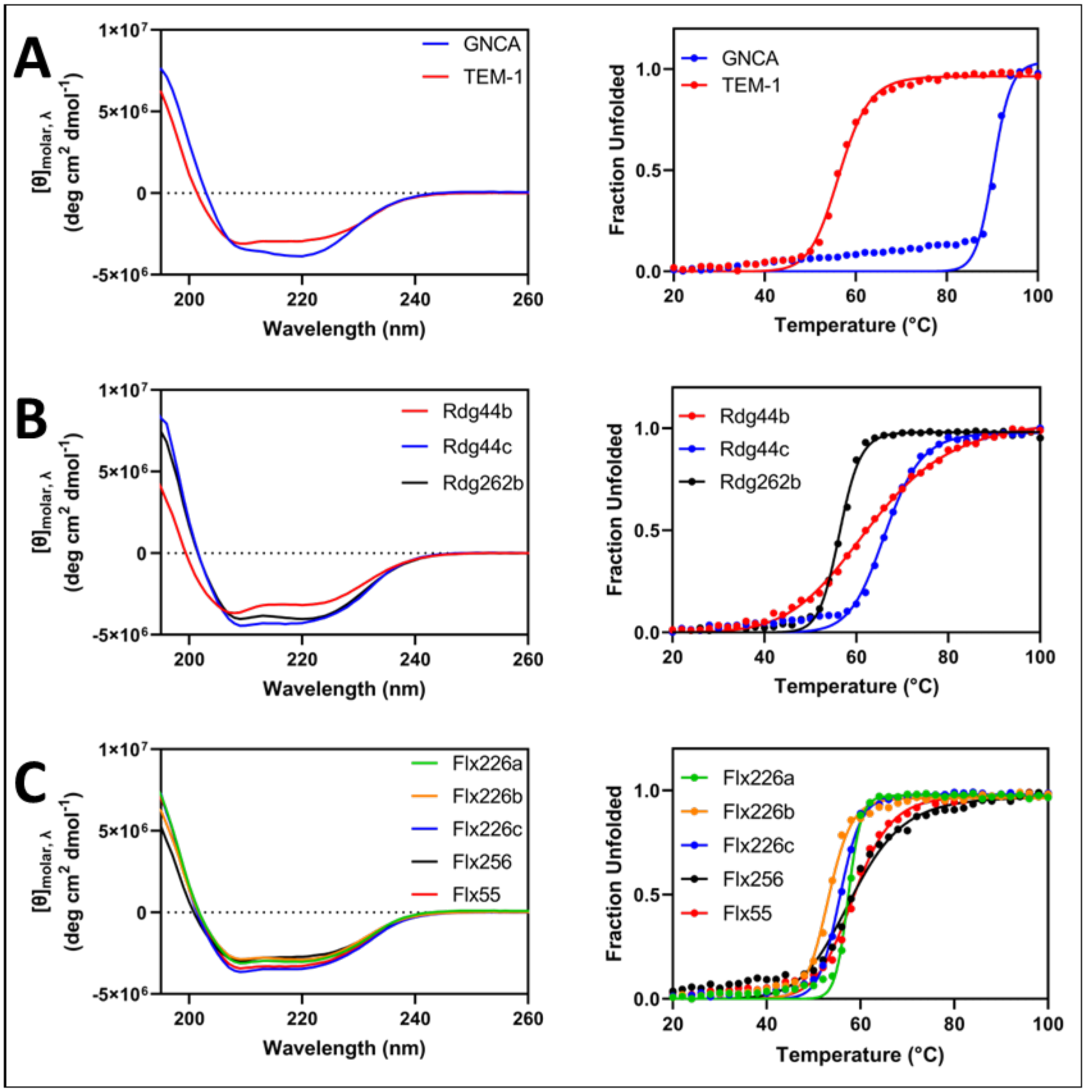

Supplementary Figure 3. Far-ultraviolet circular dichroism wavelength scans and thermal melts with fitted curves of (A) wild type GNCA and TEM-1 (B) protein designs targeting rigid residues and (C) protein designs targeting flexible residues. All measurements were performed in triplicate on a Jasco J-815 spectrophotometer and adjusted for protein concentration. Thermal melts were monitored by the absorption signal at 222 nm with a temperature slope of 5 °C/min. For wavelength scans and thermal melts, the purified protein was in TBS buffer (10mM Tris 50 mM NaCl, pH 7.0) in a cuvette with a 1 mm path length. Protein concentrations were calculated in triplicate using the absorbance at 280 nm and ranged between 0.18-0.25 mg/mL for all scans.

Supplementary Table 1. Mutations present in the computationally designed proteins and the distance of the nearest mutation to a catalytic residue in angstroms.

Designed Protein	Mutations	Distance from closest catalytic residue (Å)
Rdg44a	E37V, R43P, Y46M, F60Y, R61N, Y264M, T265M, Q278A	11.8
Rdg44b	D35K, E37V, Q39D, R43P, Y46F, F60Y, R61K, I263T, Y264M, T266S, Q278A	11.1
Rdg44c	K32V, D35Q, Q39K, R244I, T265A, R275L, Q278A	9.7
Rdg262a	Y46F, I47Q, E48L, L49T, S59Y, P62A, M182Q, V184K, T188Q, D233C, Y264F	3.9
Rdg262b	Y46F, E48L, P62A, M182T, V184R, I246Q, I246M	5.8
Flx226a	D254N, I287F	21.0
Flx226b	A227P, L250F, D254N, K256V, S258T, I287F, W290Y	12.1
Flx226c	A227P, D254N, K256V, I287F	17.5
Flx256	E212A, A227P, A249S, D254N	9.0
Flx55	E48L, L51A, N52D, S59A, V184R, T188E, T195L, A249M	9.8

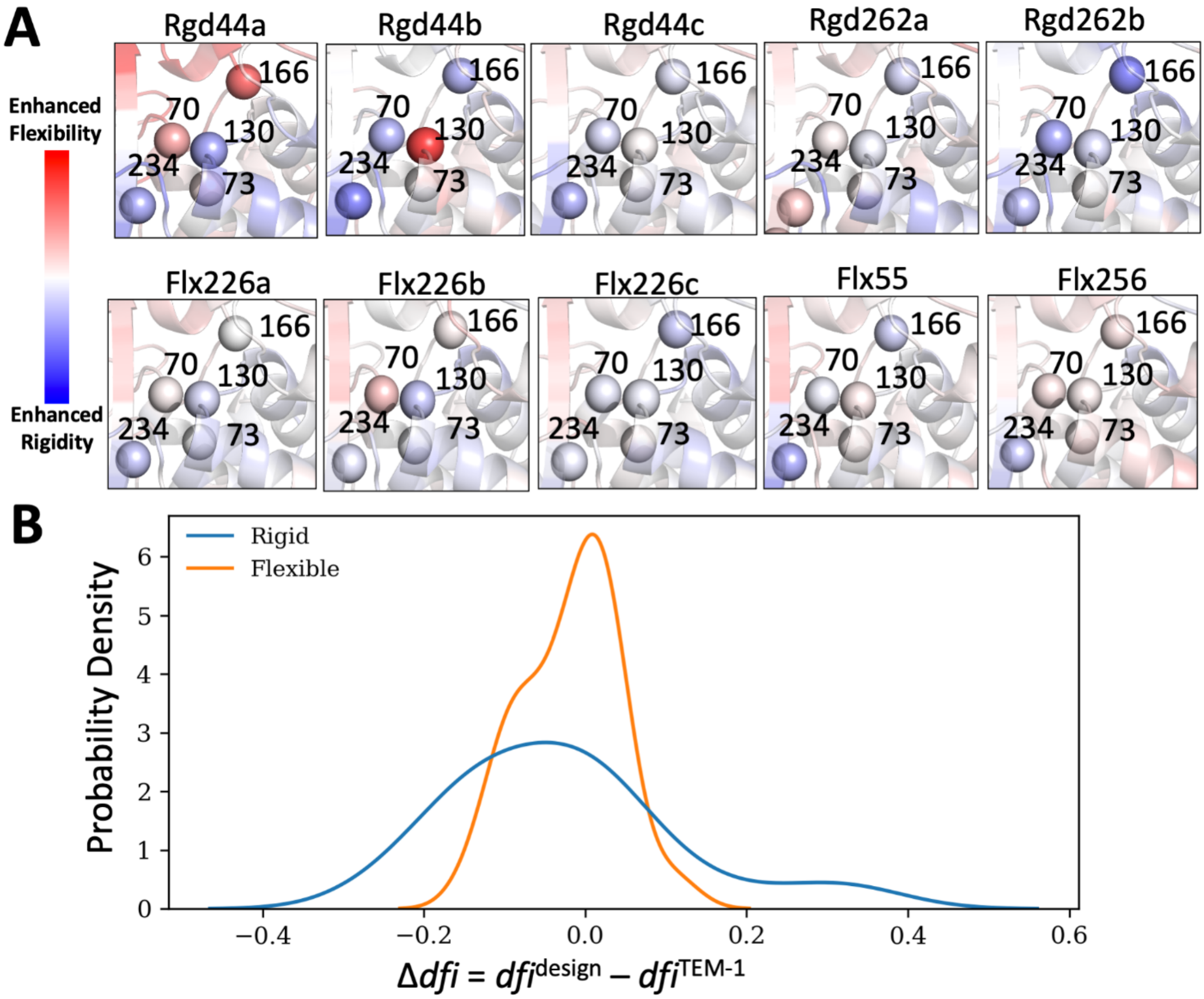

Supplementary Figure 4. The change in dynamics as measured by the Δ*dfi* mapped onto the catalytic residues of each experimentally characterized protein. A) Catalytic residues are modeled as spheres and color coded by their change in *dfi* score relative to TEM-1. B) The Δ*dfi* distribution of active site residues in the flexible and rigid designs. The flexible design distribution shows a low variance compared to that of the rigid designs. A change in *dfi* score of ± 0.2 is noteworthy as it is indicative of a shift in flexibility. This analysis suggests that designing new interactions around a rigid residue that is dynamically coupled to the active site can allosterically modulate the flexibility/rigidity of the amino acids in the active site.

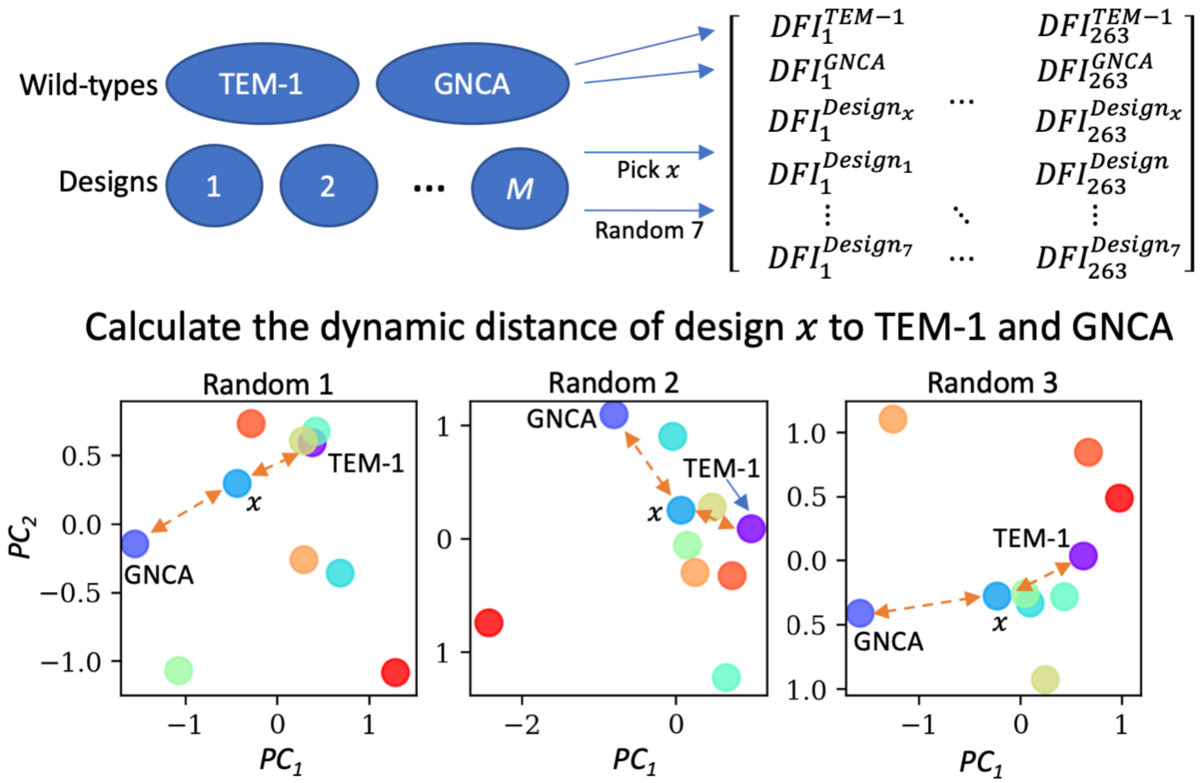

Supplementary Figure 5. Schematic of the dynamic distance calculation process. The dynamic profile of each design (using the *dfi* metric) is clustered using PCA in a set composed of TEM-1, GNCA, and seven randomly chosen designs. The dynamic distance of the design from TEM-1 and GNCA is calculated. Notably, the dynamic distance of the designed protein from TEM-1 and GNCA varies according to the set of proteins incorporated. To capture a statistically accurate distribution, this procedure is iterated a thousand times, each time varying the set of designed proteins.

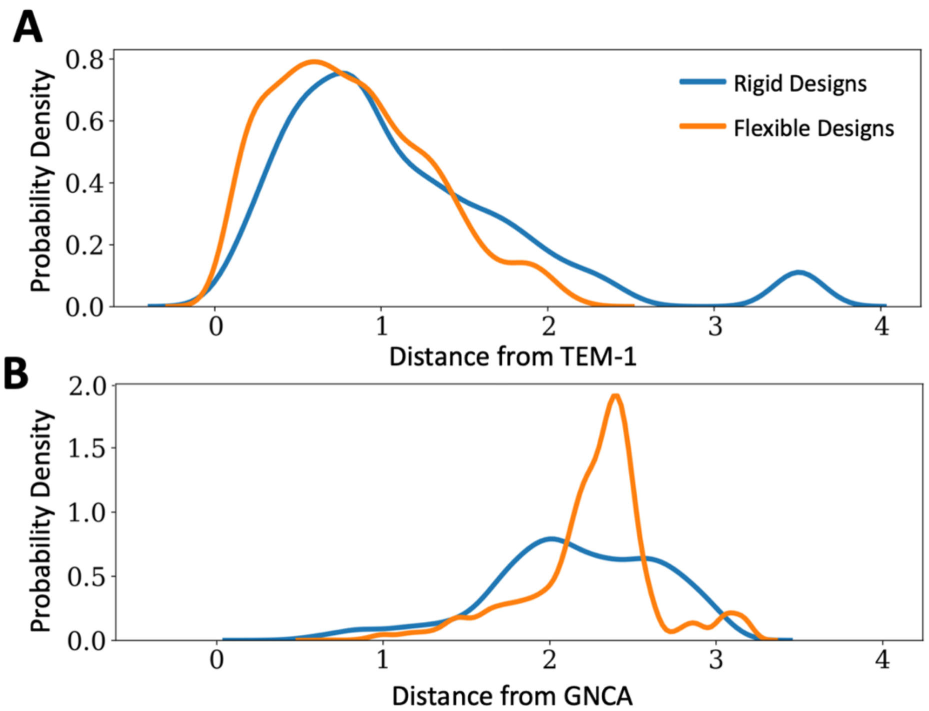

Supplementary Figure 6. Dynamic distance distribution from (A) TEM-1 and (B) GNCA for all experimentally characterized rigid (blue) and flexible designed proteins (orange). The distribution of the rigid designs shows a displacement moving away from TEM-1 and closer to GNCA. Inversely, the uncoupled flexible designs form a narrow distribution close to TEM-1 and further away from GNCA.

Detailed Rosetta methods

All calculations were carried out using Rosetta version: 442bff4fb7bf2ccb44655e8d15276c9bccfbbd0. The following command line was used to minimize the total energy of the 1btl crystal structure from the Protein Data Bank using the Rosetta relax protocol:

```
<Path to>/Rosetta/main/source/bin/relax.default.linuxgccrelease -s
<input_file> @<path to>/relax.flags
```

The contents of relax.flags was:

```
-nstruct 1
-relax:default_repeats 5
-relax:constrain_relax_to_start_coords
-relax:coord_constrain_sidechains
-relax:ramp_constraints false
-ex1
-ex2
-use_input_sc
-flip_HNQ
-ignore_unrecognized_res
-relax:coord_cst_stdev 0.5
```

The DesignAround protocol was initiated with the following command line:

```
<path to>/Rosetta/main/source/bin/rosetta_scripts.linuxgccrelease -
out:nstruct 25 -jd2:ntrials 50 -parser:protocol <path to>/design.xml -
packing:resfile <path to>[resfile] -database <path to>/Rosetta/main/database
-out::overwrite -s <input file>
@<path to>/general_design.flags
```

Where the contents of general_design.flags was:

```
-run:preserve_header
-output_virtual true
-use_input_sc
-no_his_his_pairE
-score::hbond_params correct_params
-lj_hbond_hdis 1.75
-lj_hbond_OH_donor_dis 2.6
-linmem_ig 10
-nblist_autoupdate true
-in:ignore_unrecognized_res
-out::overwrite
```

And the contents of design.xml was:

```
<ROSETTASCRIPTS>
  <SCOREFXNS>
     <ScoreFunction name="ref2015" weights="ref2015.wts"/>
  </SCOREFXNS>


 <TASKOPERATIONS>
   <ReadResfile name="read_res" filename<path_to_resfile>"/>
   <DesignAround name="des_aro" design_shell="<desired_design_sphere>"
resnums="<target_residue>" repack_shell="<design_sphere+4>" allow_design="1"
resnums_allow_design="0"/>
  </TASKOPERATIONS>

  <MOVERS>
    <PackRotamersMover name="prm" scorefxn="ref2015"
task_operations="des_aro,read_res"/>

    <MinMover name="min" scorefxn="ref2015" chi="1" bb="0" jump="ALL"
type="dfpmin_armijo_nonmonotone" tolerance="0.001" max_iter="1000"/>

    <MinMover name="min_bb" scorefxn="ref2015" chi="1" bb="1" jump="ALL"
type="dfpmin_armijo_nonmonotone" tolerance="0.001" max_iter="1000"/>

    <GenericMonteCarlo name="multi_min" mover_name="min_bb"
scorefxn_name="ref2015" trials="10" sample_type="low" temperature="0.6"
drift="0" recover_low="1" preapply="0"/>

    </MOVERS>

  <PROTOCOLS>
    <Add mover_name="prm"/>
    <Add mover_name="multi_min"/>
    <Add mover_name="prm"/>
```

```
  </PROTOCOLS>

</ROSETTASCRIPTS>
```

The content of the resfile was:

```
ALLAA EX 1 EX 2 USE_INPUT_SC
start
#1       A        PIKAA    H
2        A        PIKAA    P
#3       A        PIKAA    E
#4       A        PIKAA    T
#5       A        PIKAA    L
#6       A        PIKAA    V
#7       A        PIKAA    K
#8       A        PIKAA    V
#9       A        PIKAA    K
#10      A        PIKAA    D
#11      A        PIKAA    A
#12      A        PIKAA    E
#13      A        PIKAA    D
#14      A        PIKAA    Q
#15      A        PIKAA    L
#16      A        PIKAA    G
#17      A        PIKAA    A
#18      A        PIKAA    R
19       A        PIKAA    V #rigid resi
20       A        PIKAA    G #rigid resi
#21      A        PIKAA    Y
#22      A        PIKAA    I
#23      A        PIKAA    E
#24      A        PIKAA    L
#25      A        PIKAA    D
#26      A        PIKAA    L
#27      A        PIKAA    N
#28      A        PIKAA    S
#29      A        PIKAA    G
#30      A        PIKAA    K
#31      A        PIKAA    I
#32      A        PIKAA    L
#33      A        PIKAA    E
#34      A        PIKAA    S
#35      A        PIKAA    F
#36      A        PIKAA    R
37       A        PIKAA    P
#38      A        PIKAA    E
#39      A        PIKAA    E
#40      A        PIKAA    R
#41      A        PIKAA    F
42       A        PIKAA    P
#43      A        PIKAA    M
#44      A        PIKAA    M
45       A        PIKAA    S #Active site
#46      A        PIKAA    T
#47      A        PIKAA    F
48       A        PIKAA    K #Active site
#49      A        PIKAA    V
#50      A        PIKAA    L
51       A        PIKAA    L #rigid resi
#52      A        PIKAA    C
#53      A        PIKAA    G
#54      A        PIKAA    A
#55      A        PIKAA    V
#56      A        PIKAA    L
#57      A        PIKAA    S
#58      A        PIKAA    R
#59      A        PIKAA    I
```

```
#60      A        PIKAA    D
#61      A        PIKAA    A
#62      A        PIKAA    G
#63      A        PIKAA    Q
#64      A        PIKAA    E
#65      A        PIKAA    Q
#66      A        PIKAA    L
#67      A        PIKAA    G
#68      A        PIKAA    R
#69      A        PIKAA    R
#70      A        PIKAA    I
#71      A        PIKAA    H
#72      A        PIKAA    Y
#73      A        PIKAA    S
#74      A        PIKAA    Q
#75      A        PIKAA    N
#76      A        PIKAA    D
#77      A        PIKAA    L
#78      A        PIKAA    V
#79      A        PIKAA    E
#80      A        PIKAA    Y
#81      A        PIKAA    S
82       A        PIKAA    P
#83      A        PIKAA    V
#84      A        PIKAA    T
#85      A        PIKAA    E
#86      A        PIKAA    K
#87      A        PIKAA    H
#88      A        PIKAA    L
#89      A        PIKAA    T
#90      A        PIKAA    D
#91      A        PIKAA    G
#92      A        PIKAA    M
#93      A        PIKAA    T
#94      A        PIKAA    V
#95      A        PIKAA    R
#96      A        PIKAA    E
97       A        PIKAA    L #rigid resi
#98      A        PIKAA    C
#99      A        PIKAA    S
#100     A        PIKAA    A
#101     A        PIKAA    A
#102     A        PIKAA    I
#103     A        PIKAA    T
#104     A        PIKAA    M
105      A        PIKAA    S #Active site
#106     A        PIKAA    D
107      A        PIKAA    N #Active site
#108     A        PIKAA    T
#109     A        PIKAA    A
#110     A        PIKAA    A
#111     A        PIKAA    N
#112     A        PIKAA    L
#113     A        PIKAA    L
#114     A        PIKAA    L
#115     A        PIKAA    T
#116     A        PIKAA    T
#117     A        PIKAA    I
#118     A        PIKAA    G
#119     A        PIKAA    G
120      A        PIKAA    P
#121     A        PIKAA    K
#122     A        PIKAA    E
#123     A        PIKAA    L
#124     A        PIKAA    T
#125     A        PIKAA    A
#126     A        PIKAA    F
#127     A        PIKAA    L
#128     A        PIKAA    H
#129     A        PIKAA    N
#130     A        PIKAA    M
```

```
#131      A         PIKAA     G
#132      A         PIKAA     D
#133      A         PIKAA     H
#134      A         PIKAA     V
#135      A         PIKAA     T
#136      A         PIKAA     R
#137      A         PIKAA     L
#138      A         PIKAA     D
#139      A         PIKAA     R
#140      A         PIKAA     W
141       A         PIKAA     E #Active site
142       A         PIKAA     P #This proline is really important for folding stability
#143      A         PIKAA     E
#144      A         PIKAA     L
#145      A         PIKAA     N
#146      A         PIKAA     E
#147      A         PIKAA     A
#148      A         PIKAA     I
149       A         PIKAA     P
#150      A         PIKAA     N
#151      A         PIKAA     D
#152      A         PIKAA     E
#153      A         PIKAA     R
#154      A         PIKAA     D
#155      A         PIKAA     T
#156      A         PIKAA     T
#157      A         PIKAA     M
158       A         PIKAA     P
#159      A         PIKAA     V
#160      A         PIKAA     A
#161      A         PIKAA     M
#162      A         PIKAA     A
#163      A         PIKAA     T
#164      A         PIKAA     T
#165      A         PIKAA     L
#166      A         PIKAA     R
#167      A         PIKAA     K
#168      A         PIKAA     L
#169      A         PIKAA     L
#170      A         PIKAA     T
#171      A         PIKAA     G
#172      A         PIKAA     E
#173      A         PIKAA     L
#174      A         PIKAA     L
#175      A         PIKAA     T
#176      A         PIKAA     L
#177      A         PIKAA     A
#178      A         PIKAA     S
#179      A         PIKAA     R
#180      A         PIKAA     Q
#181      A         PIKAA     Q
#182      A         PIKAA     L
#183      A         PIKAA     I
#184      A         PIKAA     D
#185      A         PIKAA     W
#186      A         PIKAA     M
#187      A         PIKAA     E
#188      A         PIKAA     A
#189      A         PIKAA     D
#190      A         PIKAA     K
#191      A         PIKAA     V
#192      A         PIKAA     A
#193      A         PIKAA     G
194       A         PIKAA     P
#195      A         PIKAA     L
#196      A         PIKAA     L
#197      A         PIKAA     R
#198      A         PIKAA     S
#199      A         PIKAA     A
#200      A         PIKAA     L
201       A         PIKAA     P
```

```
#202      A         PIKAA     A
#203      A         PIKAA     G
#204      A         PIKAA     W
#205      A         PIKAA     F
#206      A         PIKAA     I
#207      A         PIKAA     A
#208      A         PIKAA     D
209       A         PIKAA     K #Active site
#210      A         PIKAA     S
#211      A         PIKAA     G
#212      A         PIKAA     A
#213      A         PIKAA     G
#214      A         PIKAA     E
#215      A         PIKAA     R
#216      A         PIKAA     G
#217      A         PIKAA     S
218       A         PIKAA     R #Active site
#219      A         PIKAA     G
#220      A         PIKAA     I
#221      A         PIKAA     I
#222      A         PIKAA     A
#223      A         PIKAA     A
#224      A         PIKAA     L
#225      A         PIKAA     G
226       A         PIKAA     P
#227      A         PIKAA     D
#228      A         PIKAA     G
#229      A         PIKAA     K
230       A         PIKAA     P
#231      A         PIKAA     S
#232      A         PIKAA     R
#233      A         PIKAA     I
#234      A         PIKAA     V
235       A         PIKAA     V #rigid resi
#236      A         PIKAA     I
#237      A         PIKAA     Y
#238      A         PIKAA     T
#239      A         PIKAA     T
#240      A         PIKAA     G
#241      A         PIKAA     S
#242      A         PIKAA     Q
#243      A         PIKAA     A
#244      A         PIKAA     T
#245      A         PIKAA     M
#246      A         PIKAA     D
#247      A         PIKAA     E
#248      A         PIKAA     R
#249      A         PIKAA     N
#250      A         PIKAA     R
#251      A         PIKAA     Q
#252      A         PIKAA     I
#253      A         PIKAA     A
#254      A         PIKAA     E
#255      A         PIKAA     I
#256      A         PIKAA     G
#257      A         PIKAA     A
#258      A         PIKAA     S
#259      A         PIKAA     L
#260      A         PIKAA     I
#261      A         PIKAA     K
#262      A         PIKAA     H
#263      A         PIKAA     W
```

Sequences of Designed Proteins in FASTA format

>Native β-lactamase signal peptide
MSIQHFRVALIPFFAAFCLPVFA
>Rdg262a

HPETLVKVKDAEDQLGARVGFQLTDLNSGKILEYFRAEERFPMMSTFKVLLCGAVLSRIDAGQEQLGR
RIHYSQNDLVEYSPVTEKHLTDGMTVRELCSAAITMSDNTAANLLLTTIGGPKELTAFLHNMGDHVTR
LDRWEPELNEAIPNDERDTTQPKAMAQTLRKLLTGELLTLASRQQLIDWMEADKVAGPLLRSALPAG
WFIACKSGAGERGSRGIIAALGPDGKPSRIVVIFTTGSQATMDERNRQIAEIGASLIKHW
>Rdg262b
HPETLVKVKDAEDQLGARVGFILLDLNSGKILESFRAEERFPMMSTFKVLLCGAVLSRIDAGQEQLGRRI
HYSQNDLVEYSPVTEKHLTDGMTVRELCSAAITMSDNTAANLLLTTIGGPKELTAFLHNMGDHVTRL
DRWEPELNEAIPNDERDTTTPRAMATTLRKLLTGELLTLASRQQLIDWMEADKVAGPLLRSALPAGWF
IADKSGAGERGSRGQIAALGPDGKPSRIVVIMTTGSQATMDERNRQIAEIGASLIKHW
>Rdg44a
HPETLVKVKDAVDQLGAPVGMIELDLNSGKILESYNPEERFPMMSTFKVLLCGAVLSRIDAGQEQLGRR
IHYSQNDLVEYSPVTEKHLTDGMTVRELCSAAITMSDNTAANLLLTTIGGPKELTAFLHNMGDHVTRL
DRWEPELNEAIPNDERDTTMPVAMATTLRKLLTGELLTLASRQQLIDWMEADKVAGPLLRSALPAGW
FIADKSGAGERGSRGIIAALGPDGKPSRIVVIMMTGSQATMDERNRAIAEIGASLIKHW
>Rdg44b
HPETLVKVKKAVDDLGAPVGFIELDLNSGKILESYKPEERFPMMSTFKVLLCGAVLSRIDAGQEQLGRRI
HYSQNDLVEYSPVTEKHLTDGMTVRELCSAAITMSDNTAANLLLTTIGGPKELTAFLHNMGDHVTRL
DRWEPELNEAIPNDERDTTMPVAMATTLRKLLTGELLTLASRQQLIDWMEADKVAGPLLRSALPAGW
FIADKSGAGERGSRGIIAALGPDGKPSRIVVTMTSGSQATMDERNRAIAEIGASLIKHW
>Rdg44c
HPETLVVVKQAEDKLGARVGYIELDLNSGKILESFRPEERFPMMSTFKVLLCGAVLSRIDAGQEQLGRRI
HYSQNDLVEYSPVTEKHLTDGMTVRELCSAAITMSDNTAANLLLTTIGGPKELTAFLHNMGDHVTRL
DRWEPELNEAIPNDERDTTMPVAMATTLRKLLTGELLTLASRQQLIDWMEADKVAGPLLRSALPAGW
FIADKSGAGERGSIGIIAALGPDGKPSRIVVIYATGSQATMDELNRAIAEIGASLIKHW
>Flx226a
HPETLVKVKDAEDQLGARVGYIELDLNSGKILESFRPEERFPMMSTFKVLLCGAVLSRIDAGQEQLGRRI
HYSQNDLVEYSPVTEKHLTDGMTVRELCSAAITMSDNTAANLLLTTIGGPKELTAFLHNMGDHVTRL
DRWEPELNEAIPNDERDTTMPVAMATTLRKLLTGELLTLASRQQLIDWMEADKVAGPLLRSALPAGW
FIADKSGAGERGSRGIIAALGPNGKPSRIVVIYTTGSQATMDERNRQIAEIGASLFKHW
>Flx226b
HPETLVKVKDAEDQLGARVGYIELDLNSGKILESFRPEERFPMMSTFKVLLCGAVLSRIDAGQEQLGRRI
HYSQNDLVEYSPVTEKHLTDGMTVRELCSAAITMSDNTAANLLLTTIGGPKELTAFLHNMGDHVTRL
DRWEPELNEAIPNDERDTTMPVAMATTLRKLLTGELLTLASRQQLIDWMEADKVAGPLLRSALPPGW
FIADKSGAGERGSRGIIAAFGPNGVPTRIVVIYTTGSQATMDERNRQIAEIGASLFKHY
>Flx226c
HPETLVKVKDAEDQLGARVGYIELDLNSGKILESFRPEERFPMMSTFKVLLCGAVLSRIDAGQEQLGRRI
HYSQNDLVEYSPVTEKHLTDGMTVRELCSAAITMSDNTAANLLLTTIGGPKELTAFLHNMGDHVTRL
DRWEPELNEAIPNDERDTTMPVAMATTLRKLLTGELLTLASRQQLIDWMEADKVAGPLLRSALPPGW
FIADKSGAGERGSRGIIAALGPNGVPSRIVVIYTTGSQATMDERNRQIAEIGASLFKHW
>Flx256
HPETLVKVKDAEDQLGARVGYIELDLNSGKILESFRPEERFPMMSTFKVLLCGAVLSRIDAGQEQLGRRI
HYSQNDLVEYSPVTEKHLTDGMTVRELCSAAITMSDNTAANLLLTTIGGPKELTAFLHNMGDHVTRL
DRWEPELNEAIPNDERDTTMPVAMATTLRKLLTGELLTLASRQQLIDWMAADKVAGPLLRSALPPGW
FIADKSGAGERGSRGIIASLGPNGKPSRIVVIYTTGSQATMDERNRQIAEIGASLIKHW
>Flx55
HPETLVKVKDAEDQLGARVGYILLDADSGKILEAFRPEERFPMMSTFKVLLCGAVLSRIDAGQEQLGRR
IHYSQNDLVEYSPVTEKHLTDGMTVRELCSAAITMSDNTAANLLLTTIGGPKELTAFLHNMGDHVTRL

DRWEPELNEAIPNDERDTTMPRAMAETLRKLLLGELLTLASRQQLIDWMEADKVAGPLLRSALPAGW
FIADKSGAGERGSRGIIAMLGPDGKPSRIVVIYTTGSQATMDERNRQIAEIGASLIKHW
